# Post-traumatic stress disorder following patient assaults among staff members of mental health hospitals: a prospective longitudinal study

**DOI:** 10.1186/1471-244X-6-15

**Published:** 2006-04-10

**Authors:** Dirk Richter, Klaus Berger

**Affiliations:** 1 Institute of Sociology, University of Muenster, Scharnhorststrasse 121, D-48151 Muenster, Germany; 2 Westphalian Hospital Muenster, Friedrich-Wilhelm-Weber-Strasse 30, D-48147 Muenster, Germany; 3 Institute of Epidemiology and Social Medicine, Medical School, University of Muenster, Domagkstrasse 3, D-48129 Muenster, Germany

## Abstract

**Background:**

Violence by patients against staff members in mental health institutions has become an important challenge. Violent attacks may not only cause bodily injuries but can also have posttraumatic consequences with high rates of stress for mental health staff. This study prospectively assessed posttraumatic stress disorder (PTSD) in employees who were severely assaulted by patients in nine German state mental health institutions.

**Methods:**

During the study period of six months 46 assaulted staff members were reported. Each staff member was interviewed three times after the violent incident, using the Impact of Event Scale-Revised (IES-R), a widely used PTSD research tool, as well as the Posttraumatic Stress Disorder Checklist – Civilian (PCL-C).

**Results:**

In the baseline assessment following an assault by a patient, eight subjects (17%) met the criteria for PTSD. After two and six months, three and four subjects respectively still met diagnosis criteria.

**Conclusion:**

A small minority of assaulted employees suffer from PTSD for several months after a patient assault.

## Background

In recent years violence by patients against staff members in mental health institutions has become an important challenge for care providing institutions. Violent attacks may not only cause somatic injuries but can also have posttraumatic consequences with high rates of stress and other sequelae for mental health staff [1-4]. Only few studies have reported on posttraumatic stress disorder (PTSD) following a violent patient incident [5-8]. Only three of these have used an acknowledged PTSD-questionnaire [5,7,8] and only one study was longitudinal in design [5]. Aim of the present study was to assess the course of post-traumatic stress disorder among staff members of mental health hospitals after a patient assault over a time period of six months.

## Methods

Nine state mental health institutions in the state of North Rhine-Westphalia (Germany) were recruited for this study and reported patient assaults on staff members over a 6-months-period. All affected staff members who consented in written form to participate in the study were interviewed three times by trained interviewers. A baseline face-to-face-interview was conducted after the report of an assault, follow-up interviews by phone were conducted two and six months after the initial report. Median time between assault and baseline assessment was 49 days. The study was approved by the Ethics Committee of the University of Münster, Germany.

At baseline a semi-standardised questionnaire was used to collect socio-demographic data, information about the circumstances of the assault as well as emotional reactions of the assaulted staff and the support of the employee by the institution's administration. At baseline and both follow-ups additional instruments were applied. The German version [9] of the Impact of Event Scale-Revised (IES-R) [10], a widely used PTSD-research instrument, was used to measure posttraumatic stress following the assault. The German version [11] of the Posttraumatic Stress Disorder Checklist – Civilian (PCL-C) [12] was used to identify subjects with a DSM-IV diagnosis of posttraumatic stress disorder

## Results

A total of 46 assaulted staff members were reported by the nine hospitals and consented to participate in the study. Due to a confidentiality agreement with the hospitals we were not able to collect data on those staff members who declined to participate in the study. Therefore, a calculation of incidence rates of PTSD-diagnoses was not possible. Mean age of the participants was 38 years, 23 were women and the majority were nurses (70%). Other professions involved were physicians, social workers and house keeping personnel. participants had been working for an average of 13 years at the reporting mental health hospital.

After the incident, seven of the 46 participants suffered from severe physical lesions (e.g. lack of consciousness, broken bones), 28 from small to moderate physical lesions (e.g. scratches, haematomas) and 11 did not report any physical damage. At baseline PCL-C assessment revealed that 8 participants (17%) met criteria for PTSD diagnosis. We observed significant differences in overall posttraumatic stress (assessed by IES-R) according to the severity of physical lesions received during the assault (ANOVA: F = 8.5, df = 2, p = 0.001). Participants with a severe physical damage had the highest IES-R-total score (mean 64.1, SD = 13.5). However, subjects with no physical damage scored higher on the IES-R-total score (mean 48.0, SD = 16.3) than those with small or moderate physical lesions (mean 38.3, SD = 14.7). Baseline and follow-up data for IES-R-scales are provided in table 1.

**Table 1:** PTSD-diagnosis and IES-R scores at baseline, follow-up 1 and 2

	Baseline assessment		Follow-up 1	Follow-up 2	statistics*
	All subjects N = 46	Follow-up subjects N = 35	N = 35	N = 35	

PTSD	8 (17%)	6 (17%)	3 (9%)	4 (11%)	
IES-R-Total Score	44.6 (SD 17.4)	42.8 (SD 16.4)	35.4 (SD 13.7)	32.3 (SD 13.9)	p = 0.000 (df = 2, F = 12.062)
IES-R Intrusion	14.7 (SD 6.2)	14.2 (SD 6.0)	10.1 (SD 4.2)	9.3 (SD 4.1)	p = 0.000 (df = 2, F = 21.679)
IES-R Avoidance	15.3 (SD 6.1)	14.5 (SD (5.3)	11.3 (SD 5.2)	8.9 (SD 4.6)	p = 0.000 (df = 2, F = 9.846)
IES-R Arousal	14.6 (SD 6.0)	14.1 (SD 5.9)	12.8 (SD 5.1)	12.1 (SD 5.6)	p = 0.048 (df = 2, F = 3.170)

* General linear model – repeated measures

Nine individuals declined the follow-up interview, 2 additional participants rejected one part of it (the standardised questionnaires IES-R and PCL-C). These 11 individuals were excluded from the prospective analysis. The only significant difference between follow-up participants and non-participants was absence from work. Non-participants reported 26.6 days of sickness absence between the incident and baseline interview while participants reported 7.2 days (t = -2,466; df = 44, p = 0.018).

Compared to all 46 baseline participants those with a follow-up (n = 35) had the same proportion with a PTSD diagnosis, but a slightly lower IES-R total score (42.8, SD = 16.4 vs. 44.6, SD = 17.4). In this group women reported higher IES-R total scores than men at all three assessments (data not shown). At the two and six months follow-ups, the number of subjects with a PTSD diagnosis had decreased from 6 to 3 (9%) and 4 (11%), respectively. One subject who did not have a PTSD diagnosis at baseline and follow-up 1, but was severely re-assaulted afterwards, subsequently met criteria for the diagnosis at follow-up 2. No other subject reported to be re-assaulted. The IES-R-total score and all three subscores decreased over time. The decrease was significant for all scores.

## Discussion

We report the second prospective study results on the course of PTSD among assaulted staff members of mental health institutions. Up to 6 months after the assault a considerable minority of about 10% of participating employees suffer from clinically relevant posttraumatic stress. Our results are slightly higher than those reported by Wykes and Whittington, who found that 2 out of 39 subjects (5%) suffered from PTSD one month after the assault [5]. The differences might be explained by different PTSD assessment instruments and/or the small sample sizes in both studies. Our present results are also higher than those of our previous cross-sectional study which did not find any PTSD case [7]. Again, different assessment instruments were used in these studies.

As in similar studies on the course of posttraumatic stress symptoms [13] we found a significant decline in symptoms and in subjects fulfilling the criteria for a DSM-IV diagnosis of PTSD. We also found significantly higher stress symptoms in women than in men which has been reported throughout the PTSD research literature, too [14]. The reason for this difference is possibly a specific vulnerability to the PTSD-inducing effects in women. Insofar, the course of PTSD symptoms in our sample is very similar to what has generally been reported in the literature.

An important aspect of our study is that posttraumatic stress had no dose-response relation with the severity of physical damage caused by the assault in the weeks following the incident. Staff with no physical injuries reported higher stress than those who suffered from minor lesions only. As discussed in PTSD-research our findings support the view that the subjective experience of a traumatic situation is an important contributor to the amount of stress symptoms experienced later [15]. The largest decrease in mental stress was observed in the first two months after the assault. But slight further decreases occurred until six months. A small minority, however, still suffers from PTSD symptoms that fulfil the criteria for a diagnosis of PTSD after six months.

The small sample size and a high potential for selection bias are obvious limitations of this study. High rates of refusal and non-completion have been reported from studies in similar settings [5] as well as from studies on post-traumatic stress in disaster victims [16].

An important reason for selection due to non-participation might be the fear of getting stigmatized after assaults. In this case our results would overestimate the frequency of PTSD. However, non-participation might also be caused by the PTSD-symptoms itself, especially the avoidant behaviour that is associated with this disorder. In that case our findings would underestimate the incidence of PTSD. This latter hypothesis is supported by a study on non-responders after a firework disaster which found that health problems were associated with lower response rates among white Dutch citizens [16]. The possible association of avoidant behaviour and non-response poses a serious problem to PTSD research because avoidant symptoms have been found to be strong predictors of a chronic course of this disorder [13].

## Conclusion

We conclude that some staff members develop PTSD after a patient assault, and a minority of them with symptoms over a period of six months. Active measures by the institution after assaults are necessary to prevent PTSD in staff. The management of posttraumatic stress in health care institutions requires firstly a strategy to recognize and report possible psychological sequelae. In the light of the above reported and other research findings, this seems to be a difficult task because of the avoiding behaviour of affected staff. Secondly, post-incident management support has to be provided by the organization. Recent PTSD-management strategies from US-forces and UK-military stress the importance of peer support [17,18]. Similar strategies have also been suggested for nursing staff [19]. A crucial point in this regard is to provide support without applying debriefing techniques which seem to worsen the course of the acute stress of traumatized subjects [20].

The research on PTSD after a patient assault is still in its beginnings. Apart from larger sample sizes and more longitudinal studies, there are several topics which have not been researched sufficiently. It is not clear whether there are specific predictors for the onset of a posttraumatic stress disorder which are associated with the psychiatric setting or with the staff that selects into these occupations. More important, however, is the issue of how to support assaulted staff after an incident and especially how to deal with the avoidant behaviour that affected employees show after assaults. Similar to research on prevention strategies that psychiatric institutions have developed concerning the management of violence and aggression [21], there is a need for research on the prevention of posttraumatic stress in affected staff.

## Competing interests

The author(s) declare that they have no competing interests.

## Authors' contributions

DR and KB contributed both to the acquisition of data, statistical analysis and writing of the manuscript.

## Pre-publication history

The pre-publication history for this paper can be accessed here:


